# Image Inpainting Using Two-Stage Loss Function and Global and Local Markovian Discriminators

**DOI:** 10.3390/s20216193

**Published:** 2020-10-30

**Authors:** Chen Li, Kai He, Kun Liu, Xitao Ma

**Affiliations:** School of Electrical and Information Engineering, Tianjin University, Tianjin 300072, China; lichen09@tju.edu.cn (C.L.); liukun_403@tju.edu.cn (K.L.); maxt0118@tju.edu.cn (X.M.)

**Keywords:** image inpainting, deep learning, loss function, generative adversarial network

## Abstract

Image inpainting networks can produce visually reasonable results in the damaged regions. However, existing inpainting networks may fail to reconstruct the proper structures or tend to generate the results with color discrepancy. To solve this issue, this paper proposes an image inpainting approach using the proposed two-stage loss function. The loss function consists of different Gaussian kernels, which are utilized in different stages of network. The use of our two-stage loss function in coarse network helps to focus on the image structure, while the use of it in refinement network is helpful to restore the image details. Moreover, we proposed a global and local PatchGANs (GAN means generative adversarial network), named GL-PatchGANs, in which the global and local markovian discriminators were used to control the final results. This is beneficial to focus on the regions of interest (ROI) on different scales and tends to produce more realistic structural and textural details. We trained our network on three popular datasets on image inpainting separately, both Peak Signal to Noise ratio (PSNR) and Structural Similarity (SSIM) between our results, and ground truths on test images show that our network can achieve better performance compared with the recent works in most cases. Besides, the visual results on three datasets also show that our network can produce visual plausible results compared with the recent works.

## 1. Introduction

Image inpainting is to restore the complete visual effects by generating the alternate structures and textures in the missing areas of images. It is an important part of many image editing operations, such as image target removal, image restoration, and image denoising [1,2,3]. The quality of filling damaged region determines the final image inpainting result, and the main challenge of image inpainting is to generate the feasible structure and realistic texture.

Image inpainting technology has been proposed for several decades. The image inpainting algorithms can be divided into three categories: propagation-based algorithm [4,5], search-based algorithm [3,6,7], and learning-based algorithm. The first one is proposed to deal with the small object removal, such as noise, rain, and scratch, which is realized by expanding the information in the existing region to the damaged region, so it may fail to restore the image with large damaged regions. The second one is realized by searching the similar image blocks in the information area and copying them to the damaged area. However, it is usually difficult to restore the complex structure and rich details because no semantic information of image is utilized.

With the development of deep learning technology, especially the convolutional neural network (CNN) and generative adversarial network (GAN) [8], the deep learning-based algorithm [9,10,11] has been widely used to deal with more complex situations. In recent years, many works [12,13,14] treated the image inpainting as a conditional generation problem by learning the mapping function between the input corrupted image and ground truth one. These methods perform well owning to learning meaningful semantics to produce a consistent structure for the missing regions. However, these methods may fail to effectively separate structure and texture information, so the results tend to be boundary crossing smooth or texture distortion. To solve this problem, some image inpainting approaches [15,16,17,18,19] were proposed using the two-stage networks. These methods restore the proper image structure in stage 1, and then use the reconstructed information in stage 2 to generate the final result. However, these methods [18,19] are usually time-consuming owning to the pre-processing of dataset before training. Besides, two stages of networks [17,18,19] usually need to be trained separately; thus, the end-to-end training cannot be achieved and the training complexity is increased. In addition, some methods may fail to select an appropriate intermediate state, which will result in the misleading or less available information in state 2 of network.

To solve these problems, this paper proposes a two-stage loss function. For different network stages, the Gaussian convolution kernel with different parameters is utilized for image filtering. For example, in the coarse network, some image details are filtered to make the network much easy to focus on the image structure, while, in the refinement network, only noises are filtered to better recover the image details. In addition, inspired by Reference [13,20], a global and local PatchGANs structure is proposed, in which different discriminators are used to supervise the regions on different scale and achieve more realistic results.

In this paper, we improved the coarse-to-fine network [16] using our proposed two-stage loss function and GL(Global and Local)-PatchGANs. The qualitative and quantitative experiments on several public datasets demonstrate that our method achieves competitive results against the state-of-the-art ones. The main contributions of our paper are summarized as:

- A two-stage loss function is proposed for image inpainting to make the network easy to restore plausible structure in coarse network and generate vivid texture in refinement network.
- A practical patch-based GL-PatchGANs discriminator, which focuses on the generated images on different scales, is proposed to achieve the more feasible image structures and details.
- Experiments on multiple public datasets show that our method achieves competitive results compared with the state-of-the-art ones.

## 2. Related Work

### 2.1. Image Inpainting Based on Deep Learning

With the development of deep learning technology, the learning-based image inpainting algorithms have been widely used and have achieved better performance owing to the full use of semantic information of images. For example, Pathak et al. [12] applied CNN and GAN to image inpainting and used the encoder-decoder structure to extract image features and restore the damaged regions. The early image inpainting network can only restore the square damaged area, so Iizuka et al. [13] utilized global and local discriminators to supervise whole image and square restored area, respectively. Yu et al. [15] proposed the contextual attention to search for the relationship among the dispersed similar feature blocks and used different masks to represent the damaged areas; thus, the image inpainting network could restore the damaged areas with arbitrary shape. Liu et al. [14] proposed the partial convolution to overcome the deficiency of vanilla convolution in image inpainting networks, which updates the masks with the change of damaged regions. Yu et al. [16] used the soft mask to instead the binary mask in the network to better represent the restored situation of image. Besides, Xie et al. [21] reversed the masks in the encoder network and put them in the decoder network to realize the update of damaged region. In addition, Yang et al. [22] considered the structure information in image generation network to produce the realistic structural images. Yu et al. [23] proposed the region normalization for image inpainting and conducted the batch normalization in the damaged and undamaged areas, respectively.

### 2.2. Existing Two-Stage Networks

Yu et al. [15] first proposed the two-stage network using the L1 loss between the result and ground truth. At stage 1, the rough results were obtained and used as the input of stage 2 to obtain the final results. Besides, Nazeri et al. [17] proposed EdgeConnect method. At stage 1, the image contours were restored and used for the assistance of stage 2 to obtain the final result. Song et al. [18] proposed segmentation prediction and guidance network and took the image after semantic segmentation as the intermediate state of two-stage network. Ren et al. [19] first recovered the structural image by removing the high-frequency components and then used the appearance flow to obtain the final result.

These two-stage networks can gradually restore images owning to adopting the intermediate stage. However, many networks need to be trained separately, which will increase the time consuming in training process. To solve this problem, this paper proposed a two-stage loss function and used it in the coarse and fine networks, respectively.

## 3. Proposed Approach

This section details our proposed approach. First, the proposed two-stage loss function is discussed. Second, a global and local markovian discriminator, named GL-PatchGAN, is proposed. Last, an improved coarse-to-fine generative adversarial network architecture for image inpainting is presented.

### 3.1. Proposed Two-Stage Loss Function

It is very important to select the proper intermediate state in the coarse-to-fine networks, which can largely improve the quality of the restored images. Inspired by Reference [17,22], a loss function based on Sobel operator is proposed in the two phases of network in this paper.

Given the network prediction result $I_{out}$, and the ground truth image $I_{gt}$, our two-stage Sobel loss is defined as:

(1) $$I_{out}^{gauss}=G\left(I_{out}\right)\;I_{gt}^{gauss}=G\left(I_{gt}\right),$$

(2) $$I_{out}^{sobel\_y}=S_{y}\left(I_{out}^{gauss}\right)\;I_{gt}^{sobel\_y}=S_{y}\left(I_{gt}^{gauss}\right),$$

(3) $$I_{out}^{sobel\_x}=S_{x}\left(I_{out}^{gauss}\right)\;I_{gt}^{sobel\_x}=S_{x}\left(I_{gt}^{gauss}\right),$$

(4) $$L_{sobel}=∥I_{out}^{sobel\_y}-I_{gt}^{sobel\_y}{∥}_{1}+{∥I_{out}^{sobel\_x}-I_{gt}^{sobel\_x}∥}_{1},$$

where *G* is a Gaussian filtering operation for noise removal. In order to obtain the image information on different scales of two stages, different Gaussian kernels are used in our network. $S_{x}$ and $S_{y}$ are the horizontal and vertical Sobel convolution kernels, respectively. Sobel kernels have strong ability to catch the gradient of images, so they are used in our loss function to extract the image structure in coarse network and to obtain the image contour in refinement network.

In order to better recover the image structure and texture components, other edge extraction operators, such as Laplace, Robert, and Prewitt operator, are also performed in the loss function, and the comparison experiments are conducted on CelebA-HQ [24] dataset. The networks setup is identical except the operators in loss function.

As shown in Table 1, the loss function based on Sobel operator performs better than others due to the following reasons. Compared to the Sobel operator, the Robert one has smaller kernel size, as well as a smaller receptive field. The Laplace operator only uses a single convolution kernel to process images and does not extract the horizontal and vertical gradient of images, respectively. Besides, the Prewitt operator tends to smooth images when extracting image gradient. Therefore, we apply the Sobel operator on loss function in this paper for better performance.

In this paper, the proposed loss function is utilized in two stages, as well as the Gaussian convolution kernel with different sizes and standard deviations. In coarse network, the Gaussian kernel with strong filtering ability is used, while, in refinement network, the Gaussian kernel with relatively slight filtering ability is utilized. Thus, the network tends to focus on the image structure in coarse network and focus on the details of the restored image in refinement network.

**Figure 1:**
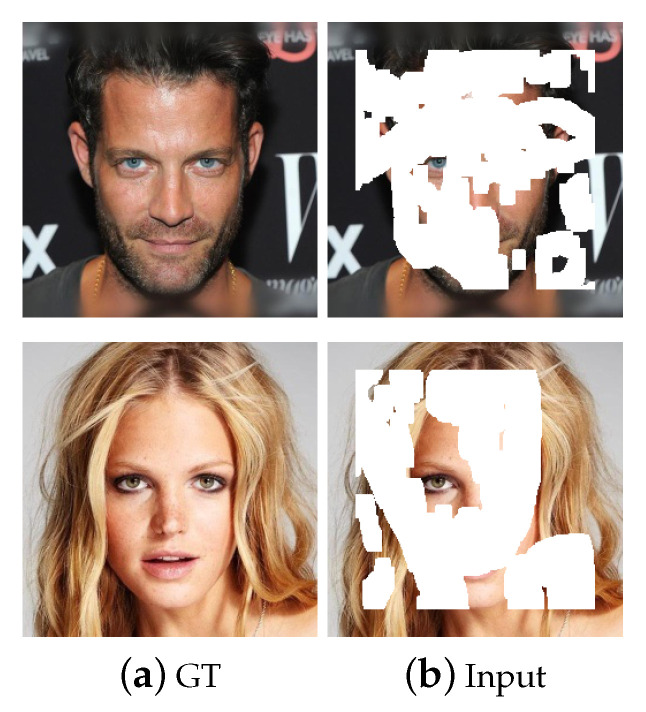

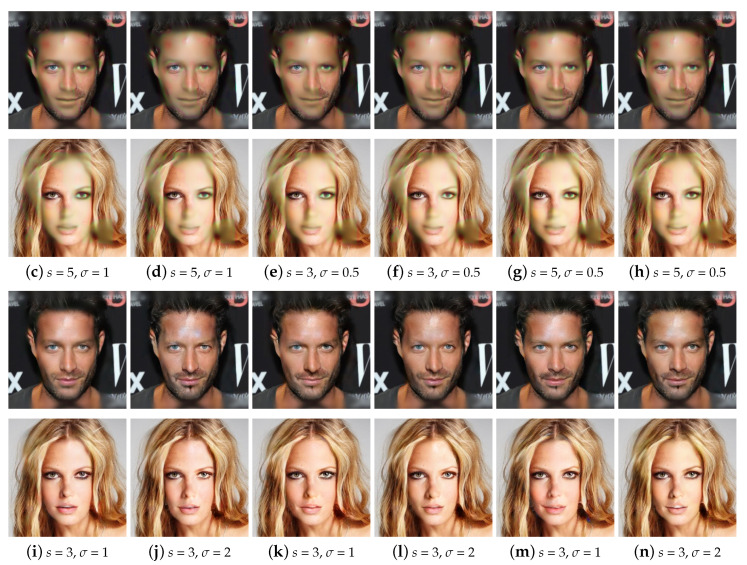
Comparison of inpainting effects at stage1 and stage 2 with different parameters.

Gaussian convolution kernels with different parameters will lead to different results. Suppose *s* and σ are the size and the standard deviation of Gaussian convolution kernel, respectively. The larger the *s* is, or the smaller the σ is, the stronger the filtering ability will be; thus, more detailed information tends to be eliminated. However, stronger filtering ability will also blur the images. To achieve the best parameters, we compared the results of convolution kernels with different sizes at different stages of network. The experiments were performed on 2000 test images taken from the dataset CelebA-HQ [24] with 10–20% irregular masks. The visual comparison results are shown in Figure 1, while the quantitatively evaluate results are shown in Table 2.

The first row of Figure 1 are the original and input images, while the second and the third row show the inpainting results at stage 1 and stage 2 with different parameters, respectively. We can see from Figure 1 and Table 1 that the best inpainting result can be achieved using the parameters *s* = 5, σ = 1 at stage 1, and *s* = 3, σ = 1 at stage 2. Therefore, the parameters above are selected in the subsequent experiments for better inpainting effects.

**Figure 2:**
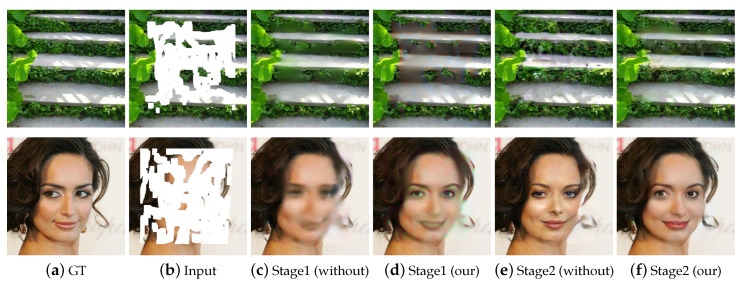
Comparison of inpainting results with or without our loss function at different stages.

To verify the effectiveness of our proposed loss function, we performed the experiments with or without it at different stages, and the results are shown in Figure 2. where Figure 2a–f are the ground truth images, input masked images, the results without and with our loss function at stage 1, and the results without and with our loss function at stage 2, respectively. We can see from Figure 2 that the network with our proposed loss function achieves better restoration effects on structural information, as shown in Figure 2c,d. The reason is that our loss function is beneficial to focus on the image contour at stage 1. In addition, the network with our proposed loss function better recovers the image details at stage 2, as shown in Figure 2e,f, because the selected Gaussian kernels in loss function is beneficial to remove slight noise and preserve the good texture images.

Given an input image with hole $I_{in}$, initial binary mask *M* (0 for holes), the network prediction $I_{out}$, and the ground truth image $I_{gt}$, our pixel reconstruction loss at two stages of network is defined as:

(5) $$L_{l1}={∥I_{out}-I_{gt}∥}_{1}.$$

Since adversarial loss is widely used to improve the visual quality of restored images, PatchGAN [25] is also used in this paper as the global discriminator $D_{g}$ and the local discriminator $D_{l}$. We denote its adversarial loss as:

(6) $$L_{D_{g}}=E_{I_{gt}}\left[logD_{g}\left(I_{gt}\right)\right]+E_{I_{comp}}log\left[1-D_{g}\left(I_{comp}\right)\right],$$

(7) $$L_{D_{l}}=E_{I_{gt}}\left[logD_{l}\left(I_{gt}\right)\right]+E_{I_{out}}log\left[1-D_{l}\left(I_{out}\right)\right],$$

(8) $$L_{D}=0.5L_{D_{g}}+0.5L_{D_{l}}.$$

The adversarial loss of our generator is defined as:

(9) $$L_{G_{g}}=E_{I_{comp}}log\left[1-D_{g}\left(I_{comp}\right)\right],$$

(10) $$L_{G_{l}}=E_{I_{out}}log\left[1-D_{l}\left(I_{out}\right)\right],$$

(11) $$L_{G}=0.5L_{G_{g}}+0.5L_{G_{l}},$$

where $I_{out}$ is the generated image, $I_{comp}$ is the image after replacing the undamaged region with those in original image, and E represents the mean of the requested item within the data range. Unlike other two-stage networks, the adversarial loss is only used on the final result in this paper; thus, the network parameters and training time can be reduced. Our final loss $L_{total}$ is defined as

(12) $$L_{total}=L_{l1}+L_{sobel}+L_{G}.$$

### 3.2. Global and Local Markovian Discriminator (Gl-Patchgan)

PatchGAN [25] was commonly used in the previous inpainting networks for filling the holes with any shapes. However, the essence of PatchGAN is to conduct the separate discriminations in different image regions with fixed size and to obtain a discriminant matrix to complete the final discrimination. The discriminator’s region of interest (ROI) in the restored image is mainly determined by the receptive fields of each neuron in the output map. The larger the discriminator’s ROI is, the easier the network tends to ignore the image detail. On the contrary, the smaller the discriminator’s ROI is, the more likely the network causes global dissonance, such as the improper eye color or the spot in a face.

**Figure 3:**
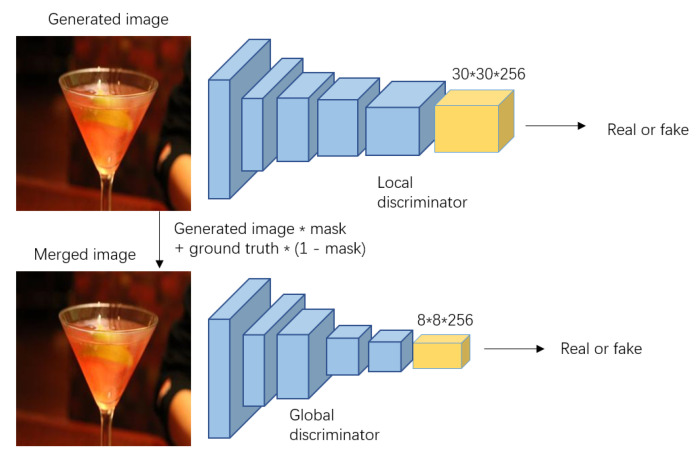
Proposed GL-PatchGAN Discriminators framework.

To solve this problem, we use the global and local discriminators of different parameters to obtain the discriminant matrices with different sizes instead. Inspired by the global and local GANs [13] and DeblurGAN-v2 [20], we improve the final merged results by using the global discriminator to produce more continuous results between the restored and undamaged regions. In addition, we deal with the whole generated image using the local discriminator to make the image details more realistic. Our proposed GL-PatchGAN discriminators are shown in Figure 3.

Table 3 compares the ablation ability of the proposed GL-PatchGAN and the original PatchGAN discriminators. In this experiment, the generator networks trained with two discriminators were tested on 2000 images taken from the dataset CelebA-HQ, and the Peak Signal to Noise ratio (PSNR) and Structural Similarity (SSIM) in terms of different masks was compared. We can see from Table 3 that the network trained with proposed GL-PatchGAN surpasses the network trained with original PatchGAN discriminators in terms of different masks, meaning that it is helpful to improve the quality of the restored images.

### 3.3. Our Model Architecture

**Figure 4:**
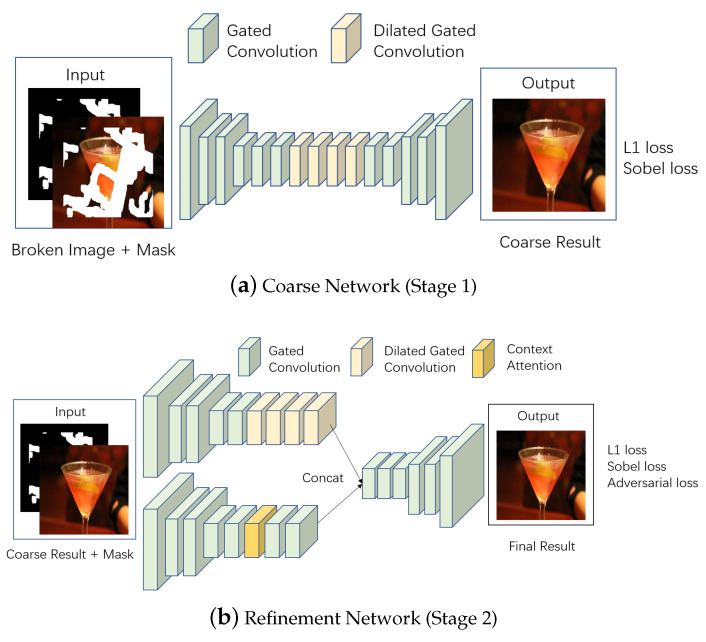
Overview of our framework.

This paper improved the coarse-to-fine network proposed by Yu [16] with the proposed two-stage loss and GL-PatchGAN loss. Our proposed framework is shown in Figure 4.

In our proposed framework, the encoder-decoder network with the proposed loss function is used in both coarse and refinement networks. As mentioned above, it is useful for the coarse network to restore the overall image structure, and is helpful for the refinement network to restore the image details. Besides, adversarial loss is applied in the refinement network to guarantee the realistic inpainting result. Using the proposed GL-PatchGAN, the discriminator tends to attach importance to both global appearance and local detail of final result.

In our coarse-to-fine network, coarse network firstly produces preliminary result with complete contour. As the skeleton of images, proper contours tend to lead to plausible final results. In refinement network, dilated convolution [26] and context attention [15] were used to search for the relationship between long distance pixels. Besides, gated convolution [16] was used in both coarse network and refinement network, which could overcome the ill-fitted for image hole-filling of vanilla convolution. However, compared to some one-stage networks, coarse-to-fine network tend to add the network parameters and training time.

Different from the existing two-stage networks, in which the separate training is necessary at different stages, our network can achieve the end-to-end training. Moreover, our network can adjust the middle inpainting state freely by changing the relative parameters; thus, the complexity of training is reduced.

## 4. Experiments

To demonstrate the effectiveness of the proposed method, we performed experiments on the internationally used datasets CelebA-HQ [24], Paris StreetView [12], and Places2 [27] with the irregular image masks provided in Reference [14], where the irregular mask dataset contains 12,000 irregular masks, in which the ratio of mask area ranges from 0–60%. The irregular mask dataset is divided into 6 sections, and each one contains 2000 mask images with the interval values of 0–10%, 10–20%, 20–30%, 30–40%, 40–50%, and 50–60%, respectively. Our model was trained on the double NVIDIA 2080TI GPUs (11GB) and implemented in tensorflow1.13. The network was trained on the 256*256 images with a batch size of 8, which were taken from the datasets CelebA-HQ [24], Paris StreetView [12], and Places2 [27], respectively. The model was optimized using Adam optimize r [28] with $\beta_{1}$ = 0.5 and $\beta_{2}$ = 0.999, and the learning rate was set to 0.0001. Our models were tested on the image with size of 256*256 on the NVIDIA 2080TI. As shown in Table 4, our model has limited network size, relative short training and inference time. Therefore, our algorithm can be used in image object removal [1,29], damaged image restoration [30,31], and other aspects.

### 4.1. Quantitative Results

Image inpainting lacks good quantitative evaluation metrics at present. Nevertheless, we compared our results with those of the latest methods on 2000 test images in CelebA-HQ and 4000 images in the test set of Places2. In the experiments, the irregular mask dataset in Reference [14] were used, and the PSNR and SSIM values were compared, as shown in Table 5 and Table 6, where CA [15] represents the generative image inpainting results with Contextual Attention (CVPR2018), PC [14] is the results of Image Inpainting for Irregular Holes Using Partial Convolutions (ECCV2018), EC [17] represents the results of EdgeConnect (ICCV2019), GC [16] represents the results of Free-Form Image Inpainting with Gated Convolution (ICCV2019), LBAM [21] is the results of Learnable Bidirectional Attention Maps (ICCV2019), and RN [23] is the results of Region Normalization for Image Inpainting(AAAI2020). Among them, the data of PC comes from Reference [23,32], while others are performed using the codes or pre-trained models provided by their authors. We can see that the proposed network achieves much better metrics and surpasses the latest ones in terms of different ratios of mask area, meaning that our proposed method is more accurate.

Our results are only a little lower than those of GC [16] on the images of dataset Places2 [27] with mask of 30–50%. The reason is probably that our two-stage loss function pays more attention to the image structure. However, the structures of some natural scenery images in Places2 [27] are too abundant and not obvious.

### 4.2. Qualitative Comparisons

**Figure 5:**
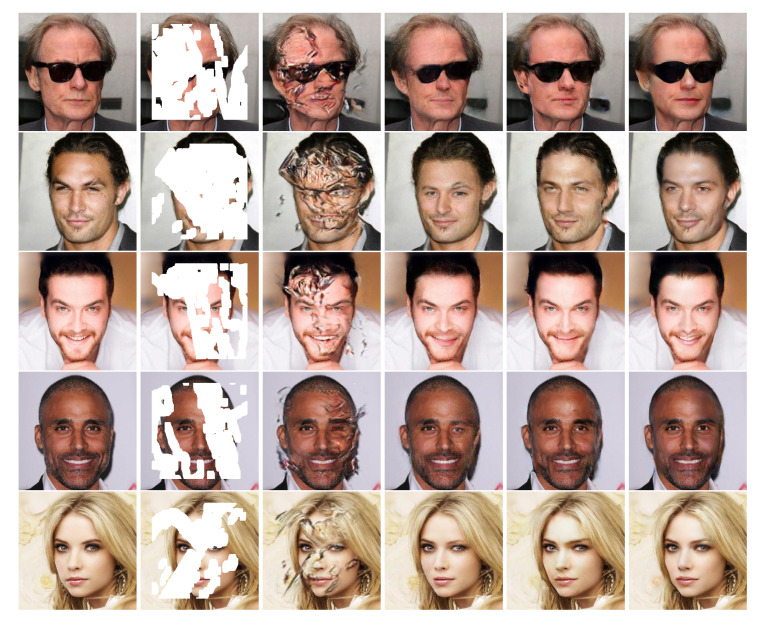

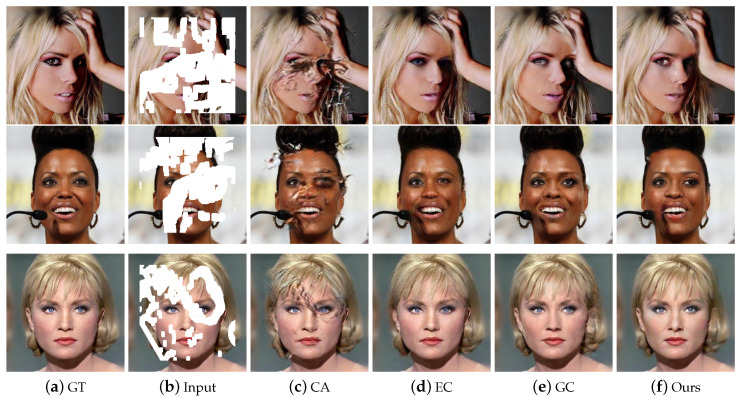
Qualitative comparisons of different methods on CelebA-HQ [24] with irregular masks.

**Figure 6:**
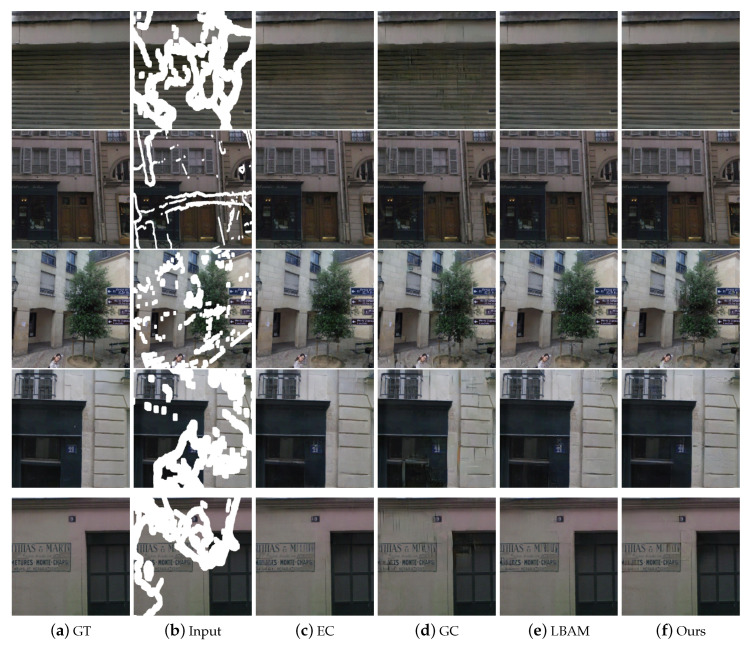
Qualitative comparisons of different methods on Paris StreetView [12] with irregular masks.

**Figure 7:**
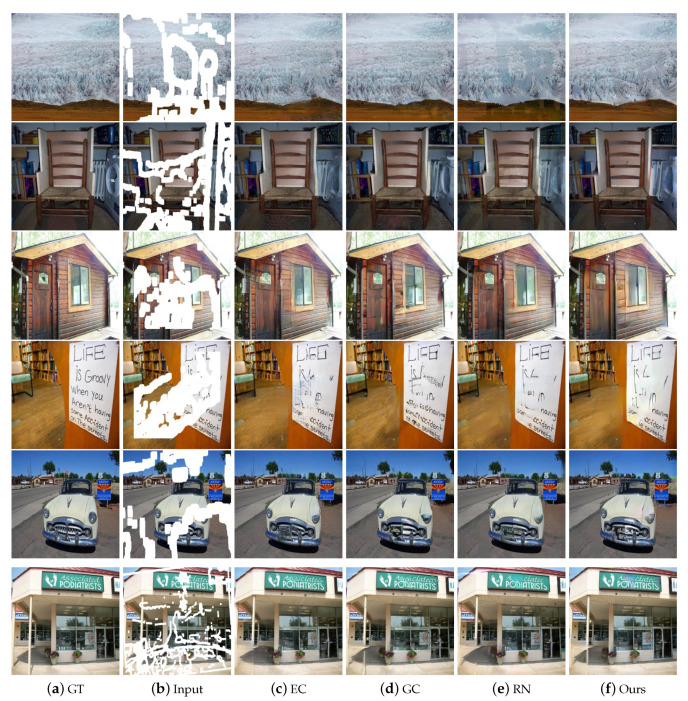
Qualitative comparisons of different methods on Places2 [27] with irregular masks.

To verify the effectiveness of the proposed method, we compared our results with several state-of-the-art ones on the datasets CelebA-HQ [24], Paris StreetView [12], and Places2 [27], respectively. Figure 5, Figure 6 and Figure 7 show the automatic inpainting results of different methods on some representative images. For all the learning-based methods, no post-processing was performed to ensure fairness. We can see that, although CA [15] can copy the feature block from the undamaged area to the damaged area in some cases, it does not pay much attention to the continuity of images. Besides, EC [17] produces poor contour at stage 1, which further leads to the poor final results. GC [16] and LBAM [21] can get smooth and plausible results; however, some artifacts still exist because the continuity of colors and lines are not well preserved. This is mainly due to the fact that these methods do not pay much attention to the structure information. Comparatively, our model performs much better and produces more visually reasonable results with feasible structure, realistic textures, and details. For example, our network produces more suitable eyes on the results of CelebA-HQ [24] dataset compared with other works. Similarly, our network also produces proper building structure and vivid textures of nature scenery on the results of Places2 [27] and Paris StreetView [12] datasets.In particular, because our proposed loss function tends to focus on the image contour in the coarse network, much plausible structure can be produced even for some complex images. Besides, with the proposed GL-PatchGANs, our method can produce realistic results with seamless boundary transitions.

### 4.3. Limitation

Since our algorithm is conducted based on deep learning, it requires relatively long training time and GPU computing power compared with the traditional algorithms. In addition, although our model achieves better effects on most images with different irregular masks, we must admit that, in order to improve the feature extraction ability, we increase the number of channels in some network modules, which will lead to the increasement of network parameters. In addition, our proposed GL-PatchGANs inevitably increases the parameters of the network. These operations will increase the training time in some degree. We hope to overcome this problem in our future works.

## 5. Conclusions

This paper proposes an end-to-end image inpainting network using the proposed two-stage loss function and GL-PatchGAN. Experimental results on the international datasets demonstrate that our two-stage loss function can bring more attention to the image structure at coarse network and restore more detailed image textures at refinement network. Besides that, our global and local PatchGANs are helpful to focus on different scales of regions and produce a more realistic structure and details in a restored image. Both quantitative and qualitative results show the superiority of our proposed method against the state-of-the-art ones.

## Figures and Tables

**Table 1:** Comparison results of networks with different operators in loss function in terms of different ratios of mask on CelebA-HQ.

	Mask (%)	Sobel	Laplace	Robert	Prewitt
PSNR${}^{+}$	10–20	32.42	31.34	31.88	31.96
	20–30	29.16	27.99	28.65	28.68
	30–40	26.77	25.63	26.38	26.31
	40–50	24.91	23.70	24.52	24.48
SSIM${}^{+}$	10–20	0.980	0.975	0.978	0.978
	20–30	0.960	0.949	0.956	0.956
	30–40	0.934	0.916	0.923	0.928
	40–50	0.901	0.875	0.894	0.895

**Table 2:** Performance comparison of two-stage loss function using Gaussian convolution kernel with different parameters at different stages.

Stage1	Stage2	PSNR${}^{+}$	SSIM${}^{+}$
without	without	30.544	0.971
*s* = 5 σ = 1	*s* = 3 σ = 1	32.420	0.980
*s* = 5 σ = 1	*s* = 3 σ = 2	32.050	0.979
*s* = 3 σ = 0.5	*s* = 3 σ = 1	32.179	0.979
*s* = 3 σ = 0.5	*s* = 3 σ = 2	32.354	0.980
*s* = 5 σ = 0.5	*s* = 3 σ = 1	32.126	0.979
*s* = 5 σ = 0.5	*s* = 3 σ = 2	32.315	0.980

**Table 3:** Comparison of results of PatchGAN and our GL-PatchGAN discriminators for different masks.

Mask Rate (%)	PatchGAN [16]		GL-PatchGAN (Ours)	
	PSNR${}^{+}$	SSIM${}^{+}$	PSNR${}^{+}$	SSIM${}^{+}$
10–20	30.512	0.968	30.544	0.971
20–30	27.272	0.940	27.305	0.944
30–40	25.043	0.906	25.073	0.910
40–50	23.246	0.864	23.280	0.870

**Table 4:** Measurement of the computational complexity of the proposed network.

Training Time			Inference Time	Network Size	FLOPs
Paris StreetView	CelebA-HQ	Places2			
1 day	2 days	3 days	19.12 ms	12M	95GFLOPs

**Table 5:** Comparison results of different methods in terms of different ratios of mask on CelebA-HQ.

	Mask(%)	CA [15]	PC [14]	EC [17]	GC [16]	LBAM [21]	Ours
PSNR${}^{+}$	10–20	25.07	31.13	30.25	31.61	30.68	32.42
	20–30	21.89	29.10	27.69	28.30	27.59	29.16
	30–40	19.69	23.46	25.55	25.96	25.36	26.77
	40–50	18.08	22.11	23.76	24.13	23.64	24.91
SSIM${}^{+}$	10–20	0.913	0.970	0.968	0.977	0.901	0.980
	20–30	0.841	0.956	0.945	0.953	0.828	0.960
	30–40	0.761	0.897	0.913	0.923	0.753	0.934
	40–50	0.675	0.839	0.871	0.887	0.673	0.901

**Table 6:** Comparison results of different methods in terms of different ratios of mask on Places2.

	Mask(%)	CA [15]	PC [14]	GC [16]	EC [17]	RN [23]	Ours
PSNR${}^{+}$	10–20	24.45	28.02	26.65	27.46	28.16	28.45
	20–30	21.14	24.90	24.79	24.53	25.06	25.12
	30–40	19.16	22.45	23.09	22.52	22.94	22.88
	40–50	17.81	20.86	21.72	20.90	21.21	21.21
SSIM${}^{+}$	10–20	0.891	0.869	0.882	0.920	0.926	0.953
	20–30	0.811	0.777	0.836	0.859	0.868	0.907
	30–40	0.729	0.685	0.782	0.794	0.804	0.852
	40–50	0.651	0.589	0.721	0.723	0.734	0.788
